# Undergraduates' perceptions on emergency remote learning in ecology in the post‐pandemic era

**DOI:** 10.1002/ece3.8659

**Published:** 2022-03-01

**Authors:** Emilio Pagani‐Núñez, Mingxiao Yan, Yixuan Hong, Yu Zeng, Sihao Chen, Peng Zhao, Yi Zou

**Affiliations:** ^1^ Department of Health and Environmental Sciences Xi’an Jiaotong‐Liverpool University Suzhou China

**Keywords:** ecology, online learning, questionnaire, remote learning, teaching methods

## Abstract

The COVID‐19 pandemic has strongly disrupted academic activities, particularly in disciplines with a strong empirical component among other reasons by limiting our mobility. It is thus essential to assess emergency remote teaching plans by surveying learners’ opinions and perceptions during these unusual circumstances. To achieve this aim, we conducted a survey during the spring semester of 2021 in an environmental science program to ascertain learners’ perceptions on online and onsite learning activities in ecology‐based modules. We were particularly interested not only in comparing the performance of these two types of activities but also in understanding the role played by learners’ perceptions about nature in shaping this pattern. Environmental science programs are rather heterogeneous from a conceptual point of view and, thus, learners may also be more diverse than in traditional ecology programs, which may affect their interest for ecology‐based modules. We assessed connectedness to nature by computing the reduced version of the Nature Relatedness Scale. Here, we found that online activities systematically obtained significantly lower scores than onsite activities regardless of the wording employed, and that altruistic behaviors were prevalent among learners. Interestingly, scores for both onsite and online activities were strongly influenced by learners’ connectedness to nature, as learners with a stronger connection to nature gave higher scores to both types of activities. Our results suggest that an effort to improve the efficacy of remote learning activities should be the focus of research about teaching methodologies in predominantly empirical scientific disciplines.

## Graphical abstract

We found that online activities systematically obtained significantly lower scores than onsite activities regardless of the wording employed, and that altruistic behaviors were prevalent among learners. Interestingly, scores for both onsite and online activities were strongly influenced by learners’ connectedness to nature, as learners with a stronger connection to nature gave higher scores to both activity types.

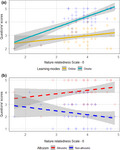

## 1 INTRODUCTION

We are living interesting times. The COVID‐19 pandemic is representing a huge challenge for learning and teaching, not only by limiting our mobility (Flaxman et al., 2020), but also by reducing available resources for education and disrupting the normal functioning of educational institutions (Daniel, 2020). This is particularly evident in disciplines with a strong empirical component, such as ecology. Traditionally, ecology is learnt discussing key concepts in the classroom and acquiring practical skills in the field and the lab. In such disciplines, a conflict between concept‐based lecturing and empirical skill acquisition is often apparent (Caulton, 1970; Openshaw & Whittle, 1993). It is necessary to reflect on our practice to ascertain to what extent these two curricular aspects, that is, concepts and skills, and the means to deliver them, are connected efficiently in teaching curriculums. The need to develop contingency remote teaching plans has elicited careful consideration of suitable online teaching tools for emergency contexts (Adedoyin & Soykan, 2020; Bozkurt et al., 2020; Hodges et al., 2020; Rapanta, 2020). This might be seen as an opportunity not only for ecological research (Rutz et al., 2020), but to review these approaches to learning in ecology and other disciplines with a strong empirical component (Bacon & Peacock, 2021).

In his seminal work, Gibbs emphasized the importance of “learning by doing” (Gibbs, 1988). A way to connect more efficiently concepts and skills would be to determine how students perceive online versus onsite learning, empirical versus normative learning activities, and transcending these disconnected frameworks (Henry, 2009). Alternative assessments, which have been object of thorough reflection since long ago (Brown et al., 1994), turn into a central element in the current context. Normative teaching frameworks emphasize activities linked to marking, in which each activity conducted in class is object of evaluation by teachers. Conversely, empirical activities, such as active learning projects (Gahl et al., 2020), can be disregarded as less efficient in motivating students into acquiring new knowledge without having to pass an exam. Fortunately, in the current critical context, developing innovative online teaching and learning approaches, and using any available technologies, has become a key priority (Gahl et al., 2020; Geange et al., 2020; Houtz et al., 2020; Richter et al., 2020; Van Haeften et al., 2020).

In this project, we surveyed learners’ perceptions on online and onsite teaching, assessed the efficacy of these two approaches in connecting concepts and skills, and determined how these patterns were influenced by connectedness to nature. Moreover, empirical activities often rely on collaborative or cooperative learning approaches, which may be perceived as unproductive or superfluous by learners. We thus assessed learners’ willingness to collect data that would be later shared with their classmates and potentially used for their coursework. Additionally, in a discipline such as ecology, perceptions on nature may mediate students’ responses to these diverse learning frameworks. The Nature Relatedness Scale (NRS) is a standard questionnaire aiming to quantify connectedness to nature (Nisbet et al., 2009). By incorporating the NRS into assessment of learning and teaching approaches, it is possible to determine how receptive learners are to online or onsite and to empirical or normative learning strategies according to their degree of connectedness to nature. Moreover, connectedness to nature is often interpreted as a measure of altruistic behaviors (Mayer & Frantz, 2004), which may be a key feature of innovative learning approaches and, thus, we were particularly interested in assessing the relationships between these two behaviors. We used the shortened version of the questionnaire designed by Nisbet and Zelenski (2013).

Here, by enquiring students about their learning experience with a questionnaire, we determined learners' perceptions on online and onsite learning and whether the linkage between practical activities and concepts was sufficiently clear. More specifically, we aimed to answer the following research questions: (1) Are online and onsite learning activities well connected to key concepts in ecology? (2) What is the perception of undergraduates on online and onsite learning activities? (3) How undergraduates perceived empirical and altruistic learning activities? (4) Was connectedness to nature (NRS) an optimal predictor of these perceptions?

## 2 METHODS

### 2.1 Study area and cohort

In this study, we conducted a survey among 49 undergraduate students between 19 and 23 years old enrolled in the Environmental Science program of the Xi'an Jiaotong‐Liverpool University (XJTLU) (Suzhou, Jiangsu Province, PR China) during the second semester of the academic year 2020–2021. Undergraduates were enrolled in different ecology modules, namely year‐2 Aquatic Field Skills (*N* = 22), year‐3 Aquatic and Urban Ecology (*N* = 16) and year‐4 Ecology in a Changing World (*N* = 11). All undergraduates but one from South Korea were Chinese nationals. We obtained ethics approval from XJTLU's University Ethics Committee (UEC) through the Educational Development Unit (EDU) and informed consent from the undergraduates before conducting the survey. Across March and early April, undergrads were engaged in different types of online and onsite activities, such as terrestrial and aquatic biodiversity monitoring, checking nest boxes, and observing recordings of animal behavior. They also experienced a fully online learning mode during the second semester of the previous academic year, so that all undergraduates were able to express informed opinions about both types of learning approaches. Most of these field activities were instrumental for undergraduates to complete their coursework, yet other activities represented an altruistic contribution to the class—any undergraduate was able to make use of the collected data.

### 2.2 The questionnaire

We conducted a survey on three main thematic areas: perceptions on online versus onsite teaching, the connection between concepts and practical activities of these two types of activities, and on altruistic data collection (Appendix 1). We employed a five‐level quantitative Likert scale to assess learners’ perceptions (Joshi et al., 2015). There was some overlap between these questions’ themes. We enquired undergraduates about to what extent they enjoyed online versus onsite activities (Q01–Q02, Q09–Q14, and Q17–Q20) and, additionally, to what extent these activities seemed connected to concepts and theories (Q01–Q02 and Q11–Q14). Furthermore, we asked undergraduates on their perceptions on normative and non‐normative activities, namely about altruistic and non‐altruistic data collection (Q15–Q16). These questions were presented in pairs to elicit learners' reflection and were shuffled to avoid stereotyped responses. Finally, we included the shortened version of the NRS questionnaire (NR‐6) (Nisbet & Zelenski, 2013), which consists of six questions (NR‐6), to assess how connectedness to nature interacted with perceptions on learning approaches and altruistic behaviors (Q03–Q08). We relied on the NRS questionnaire because we were enquiring young adults performing a high intensity program (Salazar et al., 2020).

### 2.3 Statistical analysis

All analyses were conducted in R software (R Core Team, 2021). First, we assessed the internal consistency of the questionnaire by computing Cronbach's alpha using the package *ltm* (Rizopoulos, 2006). Since several questions enquired learners about similar concepts, Cronbach's alpha score was a reliable approach to assess its consistency (Tavakol & Dennick, 2011). The questionnaire obtained a Cronbach's alpha score of 0.83, suggesting that the different items of the test were relatively consistent.

Second, we compared the scores between the different pairs of questions (Q01 vs. Q02, Q09 vs. Q10, Q11 vs. Q12, Q13 vs. Q14, Q15 vs. Q16, Q17 vs. Q18, and Q19 vs. Q20). We did so by performing pairwise t‐tests, a standard repeated‐measures approach, so each pair of answers is compared for each interviewee. We computed the average for questions about online and onsite learning separately and performed a pairwise t‐test to see if any patterns were consistent when considering all the questions together. Connectedness to nature (NRS) was calculated as the average of the NR‐6 questions for each interviewee on the assumption that higher scores would imply stronger connectedness to nature and vice versa.

Third, we computed a linear mixed‐effects (LME) model fitted with restricted maximum likelihood using the average from questions about online learning as dependent variable and the average from the NR‐6 questionnaire as independent variable. We repeated this procedure alternatively using the average from questions about onsite learning and the scores of questions Q15 and Q16 about altruistic behaviors as dependent variables. We included module (i.e., class group) as random factor. LME models were constructed using the package *nlme* (Pinheiro et al., 2007).

## 3 RESULTS

**FIGURE 1:**
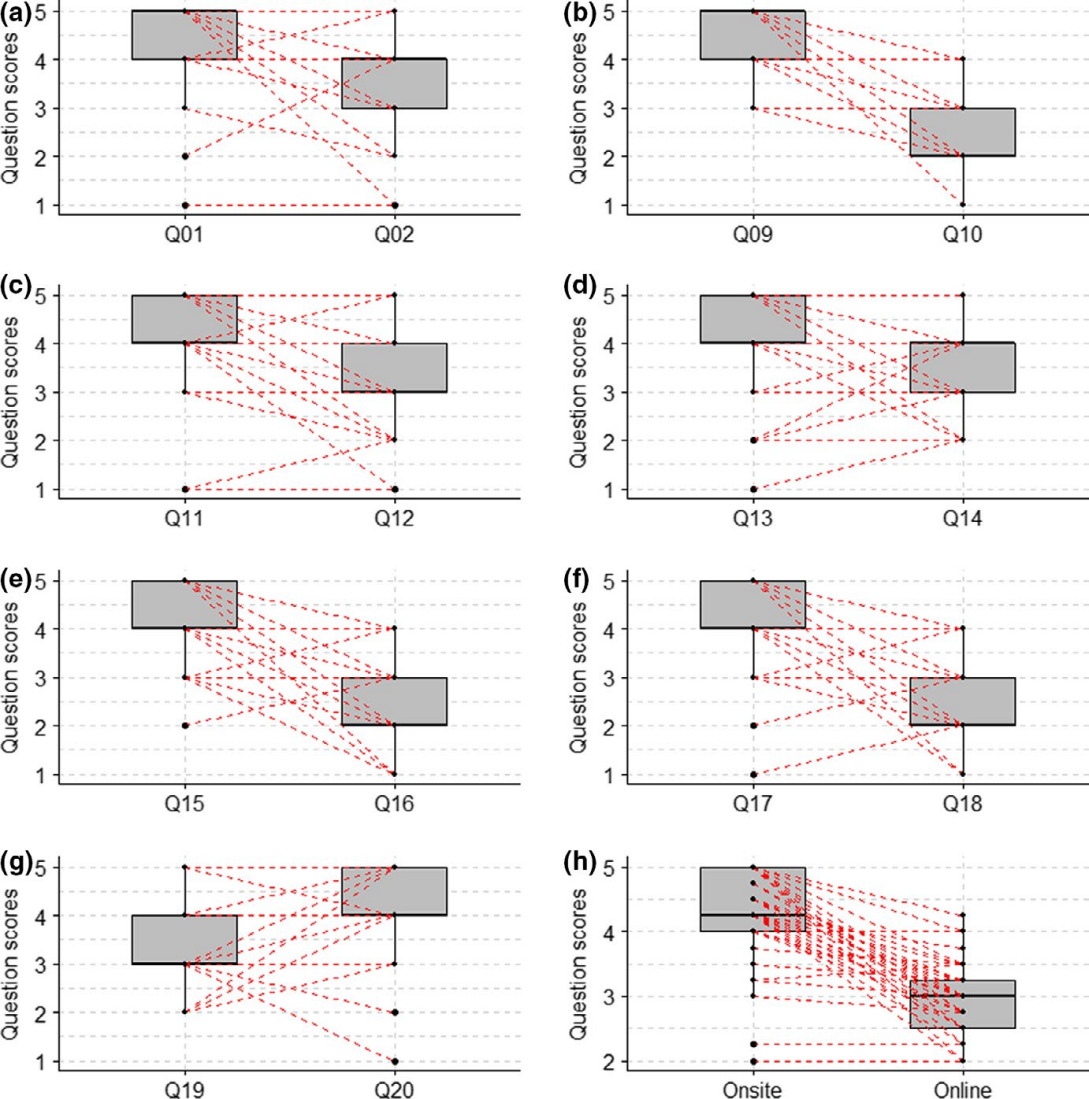
Differences in questionnaire scores between pairs of related questions (see Appendix 1 for a question list). Q01, Q09, Q11, Q13, and Q17 enquired learners about online activities, while Q02, Q10, Q12, Q14, and Q18 enquired learners about onsite activities. Q19 was about onsite and Q20 about online learning. Q15 and Q16 characterized altruistic behaviors. Online and onsite categories represent the averages of each type of questions. Thick horizontals represent average scores, thin lines represent 25% and 75% quartiles, and vertical lines represent standard deviations, while red lines connect answers of each interviewee

**FIGURE 2:**
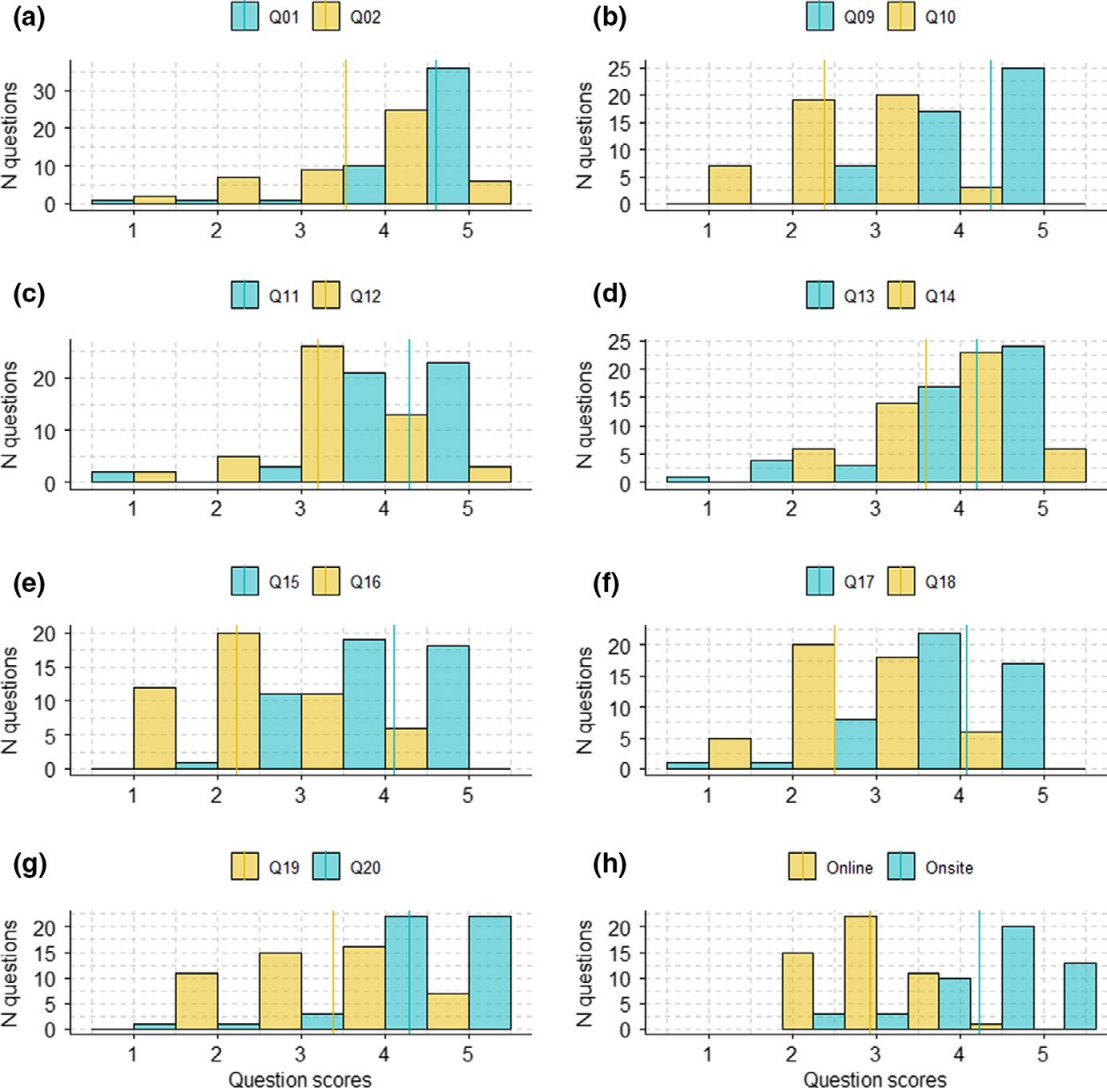
Histograms showing score distribution for each pair of related questions (see Appendix 1 for a question list). Q01, Q09, Q11, Q13, and Q17 enquired learners about online activities, while Q02, Q10, Q12, Q14, and Q18 enquired learners about onsite activities. Q19 was about onsite and Q20 about online learning. Q15 and Q16 characterized altruistic behaviors. Online and onsite categories represent the averages of each type of questions. Yellow color characterized questions about online learning and blue color characterized questions about onsite learning. Vertical lines represent questions’ averages

In all pairs of questions, undergraduates gave higher scores to onsite than to online learning (Table 1, Figures 1 and 2). More specifically, undergraduates consistently gave higher scores to the idea that ecology has a strong empirical component rather than being a discipline that can simply be learnt through textbooks (Table 1, Figures 1a and 2a) and to the idea that onsite activities were more enjoyable than online activities (Table 1, Figures 1b and 2b). Similarly, undergraduates were more in agreement with the idea that they had learnt more practical skills during onsite than online activities (Table 1, Figures 1c and 2c), and to the idea that onsite activities were better linked to the topics covered in the modules than online activities (Table 1, Figures 1d and 2d).

**TABLE 1:** Results from pairwise t‐tests assessing differences in the scores given by individual learners to pair of questions about their perceptions on online and onsite learning (*N* = 49). Q01, Q09, Q11, Q13, and Q17 enquired learners about online activities, while Q02, Q10, Q12, Q14, and Q18 enquired learners about onsite activities. Yet, in an attempt to avoid stereotyped responses, Q19 was about onsite and Q20 about online learning. Q15 and Q16 characterized altruistic behaviors. Online and onsite categories represent the averages of each type of questions (see Appendix 1)

	*t‐value*	*p*
Q01 vs. Q02	6.47	<.01
Q09 vs. Q10	11.09	<.01
Q11 vs. Q12	7.94	<.01
Q13 vs. Q14	4.13	<.01
Q15 vs. Q16	9.05	<.01
Q17 vs. Q18	8.32	<.01
Q19 vs. Q20	−5.51	<.01
Online vs. onsite	−10.22	<.01

Furthermore, undergraduates were keen to share collected data with other undergraduates (Table 1, Figures 1e and 2e) and showed higher agreement with the idea that they would like to spend more time conducting onsite activities rather than online activities (Table 1, Figures 1f and 2f). They also were more in agreement with the idea that ecology is mainly about understanding the world around us than about statistics and big data (Table 1, Figures 1g and 2g). As a result, questions about onsite learning obtained higher scores on average than questions about online learning (Table 1, Figures 1h and 2h).

**FIGURE 3:**
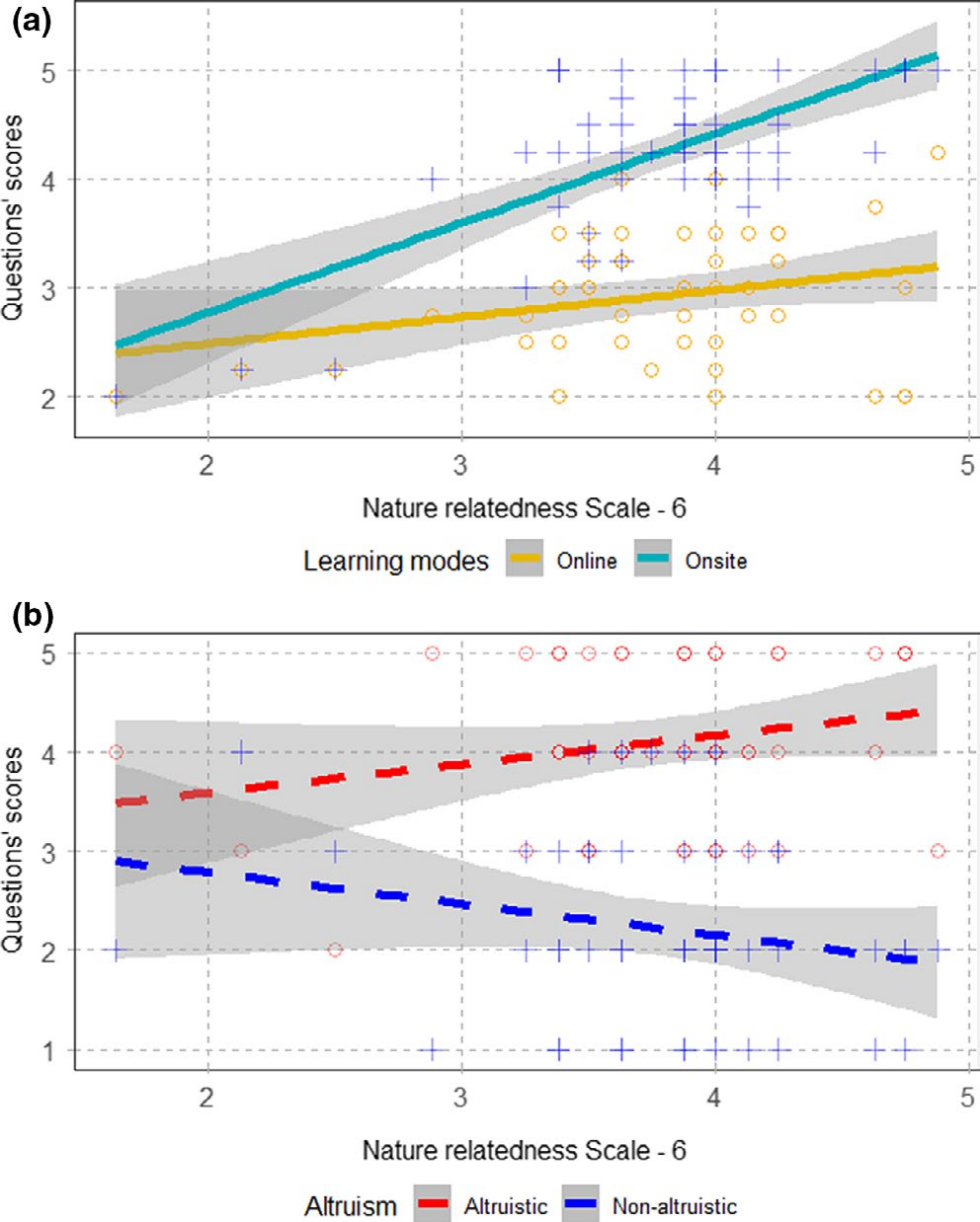
(a) Significant linear relationships (solid lines) between average NR‐6 scores per interviewee and the average for questions about online learning (empty yellow circles) and onsite learning (blue crosses). The shaded gray area represents 95% confidence intervals. (b) Non‐significant linear relationships (dashed lines) between average NR‐6 scores per interviewee and the results for questions Q15 (empty red circles) and Q16 (dark blue crosses) characterizing the degree of altruism (or lack of it, respectively) of the interviewees

Finally, undergraduates’ NR‐6 average scores correlated positively with average scores of questions about onsite learning (*β* ± *SE* = 0.30 ± 0.13, *t*_3,45_ = 2.24, *p* = .03), and with average scores of questions about online learning (*β* ± *SE* = 0.85 ± 0.12, *t*_3,45_ = 7.09, *p* < .01) (Figure 3a). Conversely, for questions regarding altruistic behaviors, we did not record significant relationships between NR‐6 and these questions’ scores (altruistic: *β* ± *SE* = 0.21 ± 0.19, *t*_3,45_ = 1.11, *p* = .27; non‐altruistic: *β* ± *SE* = −0.32 ± 0.22, *t*_3,45_ = −1.43, *p* = .16) (Figure 3b).

## 4 DISCUSSION

The current pandemic has strongly disrupted academic activities (Bacon & Peacock, 2021), particularly in developing countries (Adnan & Anwar, 2020), where there may be a scarcity of resources to deal with this situation. However, crises might also represent opportunities to improve our educational systems. For instance, out teaching practice can be improved by assessing to what extent different types of onsite and online learning activities are efficiently connected to key concepts and theories, and to assess the efficacy of these activities as learning and teaching tools (Gibbs, 1988; Hmelo, 1998; O’Mahony et al., 2012). In this study, we found support to the idea that undergraduates positively valued the key empirical component of ecology, yet this also represented a challenge in circumstances in which emergency remote learning must be implemented. Overall, undergraduates gave significantly lower scores to online than onsite activities. This may simply be a signal of the expectable frustration produced by the COVID‐19 pandemic on learners (Dilmaç, 2020), which may also be linked to a strong awareness of the gravity of the situation among Asian learners (Van et al., 2010). Yet, it may also be connected to a deeper issue with online learning and teaching approaches in strongly empirical disciplines, such as ecology. Moreover, undergraduates seemed keen to share collected data with their peers, favoring the establishment of an open learning environment based on collective efforts, which is increasingly deemed as an efficient teaching and learning approach (Ruiz‐Gallardo & Reavey, 2019).

Interestingly, NR‐6 scores were a good predictor of undergrads’ perceptions about both onsite and online learning activities. Previous studies have shown that nature connectedness is a good predictor of positive attitudes toward scientific issues and outdoor activities (Barrable & Lakin, 2020; H.‐H. Wang et al., 2020). However, in our study, students scoring higher in the NR‐6 scale gave higher scores to questions about both online and onsite learning, in spite that these questions were to some extent opposed to each other. This result suggests that undergraduates assessed their learning experience mainly based on their interest on the subject, rather than on the quality of the activity itself. Still, other studies have shown positive attitudes toward online learning, which underscores a high diversity of responses to the current pandemic (Khan et al., 2020). Acknowledging that undergraduates may have different perceptions on study subjects in different regions and circumstances could help to enhance their learning experience.

Finally, NR‐6 was disconnected from undergraduates’ perceptions on altruistic behaviors. We recorded very positive attitudes of undergraduates toward data sharing, yet this seemed to be linked to personal perceptions and behaviors rather than to the degree of connectedness to nature. A positive relationship between connectedness to nature and altruistic behaviors has been found in several studies (Barrera‐Hernández et al., 2020; Lee et al., 2015; Mayer & Frantz, 2004). Therefore, our results merit further investigation as it would be interesting to determine why this relationship is absent in our sample. National, cross‐cultural, differences in connectedness to nature and altruistic behaviors have previously been reported (Dornhoff et al., 2019; Johnson et al., 1989). In the particular case of China, where suppression of COVID‐19 has been successful (Zou et al., 2020), and a country in which the collective component of social organization is considered very important (Chen, 2000; Wang et al., 2002), undergraduates may be particularly keen to share resources regardless of their perceptions on nature.

To conclude, we found that connectedness to nature was a good predictor of positive attitudes to learning in ecology, regardless of the form in which learning and teaching was developed. Yet, we must acknowledge certain limitations of our approach. For instance, our analysis is based on self‐reported data from interviewees, which may be object to biases that are difficult to minimize. Moreover, we could have used additional nature connectedness metrics (Restall & Conrad, 2015), sample size was somewhat limited, and the social background of our interviewees was rather homogenous. Still, our results suggest that undergrads showing a stronger connection to nature were more positive about both empirical and online learning activities. Thus, promoting positive attitudes toward nature in educational programs such as ecology or environmental sciences could be a way to enhance students’ learning experience. In response to our first research question, onsite activities were better connected than online activities to key concepts in ecology. In response to our second research question, undergraduates had more positive attitudes toward onsite than online activities. Regarding our third question, undergraduates had very positive attitudes toward data sharing regardless of their degree of connectedness to nature, which may facilitate the development of collaborative research projects with a low risk of eliciting conflicts among peers. Finally, regarding the fourth research question, nature connectedness was an optimal tool to assess willingness to study ecology independently of the teaching methods employed, and it was a poor predictor of undergraduates’ altruistic behaviors.

## CONFLICT OF INTEREST

The authors have no conflict of interest to declare.

## AUTHOR CONTRIBUTIONS

**Emilio Pagani‐Núñez:** Conceptualization (lead); Data curation (equal); Formal analysis (equal); Methodology (equal); Writing – original draft (lead). **Mingxiao Yan:** Conceptualization (equal); Data curation (equal); Formal analysis (equal); Writing – review & editing (equal). **Yixuan Hong:** Conceptualization (equal); Data curation (equal); Formal analysis (equal); Writing – review & editing (equal). **Yu Zeng:** Conceptualization (equal); Data curation (equal); Formal analysis (equal); Writing – review & editing (equal). **Sihao Chen:** Conceptualization (equal); Data curation (equal); Formal analysis (equal); Writing – review & editing (equal). **Peng Zhao:** Conceptualization (equal); Data curation (equal); Formal analysis (equal); Writing – review & editing (equal). **Yi Zou:** Conceptualization (equal); Data curation (equal); Formal analysis (equal); Writing – review & editing (equal).

## Data Availability

Questionnaire data: Pagani‐Núñez, Emilio et al. (2022), Undergraduates’ perceptions on emergency remote learning in ecology in the post‐pandemic era, Dryad, Dataset, https://doi.org/10.5061/dryad.jh9w0vtd6

## APPENDIX 1

Note that the questionnaire was anonymized and thus only the question list is attached here

**Table:**

Question	Rating				
	Totally disagree	Disagree	Neutral	Agree	Totally agree
1. To learn ecological concepts, it is necessary to experience nature					
2. Ecology can be learnt using books and computers					
3. My ideal vacation spot would be a remote, wilderness area					
4. I always think about how my actions affect the environment					
5. My connection to nature and the environment is a part of my spirituality					
6. I take notice of wildlife wherever I am					
7. My relationship to nature is an important part of who I am					
8. I feel very connected to all living things and the earth					
9. Onsite fieldwork was the most enjoyable activities of the module					
10. Online activities were the most enjoyable part of the module					
11. I learnt new practical skills with onsite fieldwork					
12. I learnt new practical skills with online activities					
13. Onsite field activities are well linked to the topics covered in the module					
14. Online activities are well linked to the topics covered in the module					
15. I enjoyed collecting data that will be used by my peers					
16. I would prefer collecting my own data and not sharing it					
17. I would like to spend more time conducting field activities					
18. I would like to spend more time conducting online activities					
19. Ecology is mostly about statistics and big data					
20. Ecology is mostly about understanding the world around us					
